# The Role of the LINC01376/miR-15b-3p_R-1/FGF2 Axis in A549 and H1299 Cells EMT Induced by LMW-PAHs

**DOI:** 10.3390/toxics14010054

**Published:** 2026-01-06

**Authors:** Jiali Qin, Yamin Huang, Yixuan Hu, Haitao Ma, Zhengyi Zhang, Yuanjie Li, Shiyao Jiang, Chengyun Li, Kaikai Li, Junling Wang, Xiaoping Liu

**Affiliations:** 1 Department of Toxicology, School of Public Health, Lanzhou University, Lanzhou 730030, China; 220220913580@lzu.edu.cn (J.Q.);; 2 School of Public Health, Southeast University, Nanjing 210009, China; 3 Xiaogan Center for Disease Control and Prevention, Xiaogan 432100, China; 4 The Second Hospital & Clinical Medical School, Lanzhou University, Lanzhou 730030, China; 5 School of Clinical Medicine, Northwest Minzu University, Lanzhou 730030, China; 6 Affiliated Hospital of Northwest University for Nationalities, Lanzhou 730030, China; 7 Department of Respiratory and Critical Care Medicine, Second People’s Hospital of Gansu Province, Lanzhou 730030, China

**Keywords:** polycyclic aromatic hydrocarbons (PAHs), epithelial-mesenchymal transition (EMT), long non-coding RNA (lncRNAs), competing endogenous RNA (ceRNA), FGF2

## Abstract

Low-molecular-weight polycyclic aromatic hydrocarbons (LMW-PAHs), such as the 400 μM mixture of phenanthrene and fluorene used in this study, are prevalent environmental pollutants. Induction of epithelial–mesenchymal transition (EMT) by LMW-PAHs promote cell invasion and migration and contribute to disease pathogenesis. Long non-coding RNAs (lncRNAs) regulate gene expression by acting as competing endogenous RNAs (ceRNAs) that sequester microRNAs (miRNAs), a mechanism important for modulating EMT. Previously, regulation of the PI3K/AKT pathway and EMT in A549 cells are shown to occur through the hsa_circ_0039929/miR-15b-3p_R-1/FGF2 axis. Here, the functional role of the related LINC01376/miR-15b-3p_R-1/FGF2 axis in LMW-PAH-induced EMT was examined in A549 and H1299 cells. The miR-15b-3p_R-1 was downregulated, whereas LINC01376 and FGF2 were upregulated following LMW-PAH exposure. LINC01376 overexpression enhanced EMT, migration, and invasion. Interactions between miR-15b-3p_R-1 and FGF2, as well as direct binding of LINC01376 to miR-15b-3p_R-1, were confirmed experimentally. The results indicate that, in LMW-PAH-treated cells, LINC01376 functions as a ceRNA to sponge miR-15b-3p_R-1, thereby elevating FGF2 expression and promoting EMT, migration, and invasion. Identification of the LINC01376/miR-15b-3p_R-1/FGF2 regulatory axis highlighted as a key mechanism in LMW-PAH-driven EMT and suggests its potential as a therapeutic target in PAH-related pathologies.

## 1. Introduction

Environmental pollutants known as polycyclic aromatic hydrocarbons (PAHs) are highly carcinogenic, mutagenic, and teratogenic hazardous air pollutants characterized by high emission density and long atmospheric lifetime [1,2,3]. PAHs are categorized by molecular weight: low-molecular-weight PAHs (LMW-PAHs), such as naphthalene, fluorene, anthracene, and phenanthrene, contain two or three benzene rings; high-molecular-weight PAHs (HMW-PAHs), such as pyrene, contain four or more [4]. While most research has focused on HMW-PAHs, LMW-PAHs are the predominant PAHs bound to atmospheric particulate matter (PM) and occur at higher total air concentrations than HMW-PAHs [5]. Consequently, understanding their impact on human health is essential.

The key mechanism of pollutants toxicity is the disrupting epigenetic regulation, including gene expression networks mediated by non-coding RNAs such as long non-coding RNAs (lncRNAs) and microRNAs (miRNAs). Such disruption is recognized as a key molecular mechanism underlying pollutant-induced pathological changes in various organs, including the lung [6]. However, specific lncRNA–miRNA–mRNA regulatory axes driving LMW-PAH-induced epithelial–mesenchymal transition (EMT) a pivotal process conferring migratory and invasive properties to pulmonary cells and promoting fibrosis and cancer metastasis remain poorly characterized.

In this study, phenanthrene (Phe) and fluorene (Flu) were selected as representative LMW-PAHs due to their environmental abundance and widespread presence in particulate matter. Prior work has shown that exposure to such compounds, alone or in combination, induces significant oxidative stress and inflammatory responses in A549 lung cells. This exposure is evidenced by elevated markers including superoxide dismutase (SOD), malondialdehyde (MDA), TNF-α, and IL-6 [7,8,9]. Such early stress events are considered critical upstream triggers for pathological processes, including EMT. While PAH exposure is known to promote invasion and migration via EMT in diseases such as fibrosis and cancer [10,11], few studies have specifically examined LMW-PAH-induced EMT, and the underlying mechanisms remain largely unclear.

EMT involves the transition of epithelial cells to a mesenchymal phenotype, enabling migration, invasion, and extracellular matrix production [12]. Such shift is marked by downregulation of epithelial markers such as E-cadherin and upregulation of mesenchymal markers, including N-cadherin, vimentin, and α-smooth muscle actin (α-SMA) [13]. Key transcription factors such as Snail, Smads, ZEB1, and Twist1 orchestrate these changes [14,15,16,17,18], supported by signaling pathways like TGF-β, EGF, Wnt, ILK, RTK, and Hedgehog [19,20,21]. Increasingly, non-coding RNAs are recognized as crucial regulators of these EMT-related processes [22,23,24].

Non-coding RNAs include short species (<50 nt, miRNA, siRNA) and long non-coding RNAs (>200 nt, lncRNAs) [25]. LncRNAs are particularly abundant and, due to their diverse regulatory mechanisms, have become a major focus of epigenetic research. They modulate gene expression through transcriptional and epigenetic means, influencing processes such as proliferation, invasion, and EMT [26,27,28]. MicroRNAs, approximately 22 nucleotides in length, typically bind to the 3′UTR of target mRNAs to suppress translation or promote degradation, thereby regulating diverse physiological and pathological functions [29,30]. LncRNAs can act as competing endogenous RNAs (ceRNAs) by sequestering miRNAs, thereby derepressing miRNA target genes a mechanism increasingly implicated in EMT regulation [13,31]. The regulatory RNAs also hold promise as biomarkers for EMT-related diseases, aiding early diagnosis and treatment.

Previously, the identified hsa_circ_0039929/miR-15b-3p_R-1/FGF2 axis as a regulator of EMT in A549 cells [32]. Notably, miR-15b-3p is downregulated in conditions such as acute liver failure, while FGF2 promotes EMT, migration, and invasion [33,34]. Nevertheless, how lncRNAs interact with miR-15b-3p_R-1 and FGF2 to regulate LMW-PAH-induced EMT remains unknown. Adenocarcinoma is the most common subtype of non-small-cell lung cancer (NSCLC), accounting for approximately 70–80% of cases [35]. A549 (lung adenocarcinoma) and H1299 (lymph node metastasis-derived lung carcinoma) cell lines were selected for their established relevance in modeling NSCLC progression and PAH-induced toxicity. As widely used models, A549 is standard for studying lung-specific responses, while the more aggressive H1299 line, derived from metastasis, is particularly suited for investigating invasive phenotypes associated with EMT [36,37]. Therefore, this study aimed to investigate the role of the LINC01376/miR-15b-3p_R-1/FGF2 axis in LMW-PAH-induced EMT, and to examine how miR-15b-3p_R-1 affects cell invasion and migration in A549 and H1299 cells, thereby identifying a novel therapeutic target for PAH-associated pathologies.

## 2. Materials and Methods

### 2.1. Cell Culture and LMW-PAHs Treatment

A549 and H1299 cell lines were kindly provided by the Cancer Hospital of Gansu Province, China. Human embryonic kidney 293T cells were obtained from the School of Public Health, Lanzhou University, China. All cell lines were cultured at 37 °C in a humidified incubator with 5% CO_2_. The non-small-cell lung cancer (NSCLC) cell lines A549 and H1299 were maintained in RPMI-1640 medium (Biological Industries, Beit-Haemek, Israel). HEK 293T cells used for luciferase reporter assays due to their high transfection efficiency, were cultured in Dulbecco’s Modified Eagle Medium (DMEM; Biological Industries, Beit-Haemek, Israel). All media were supplemented with 10% fetal bovine serum (FBS) and 1% penicillin–streptomycin.

Phenanthrene (Phe, CAS: 85-01-8, 98% purity) and fluorene (Flu, CAS: 86-73-7, 98% purity) were purchased from Sigma-Aldrich (St. Louis, MO, USA). Stock solutions were prepared in dimethyl sulfoxide (DMSO) and stored at −20 °C. Based on our previous study [32], working solutions containing a 1:1 mixture of Phe and Flu were prepared in complete medium containing 10% FBS at final concentrations of 200 μM, 400 μM, and 600 μM. The selection of these three concentrations and the 24 h exposure duration was based on a systematic toxicity assessment in our prior work [7]. That study confirmed that this concentration range effectively induces target biological responses (e.g., inflammation, oxidative stress) while maintaining cell viability well above the lethal median dose, thereby ensuring that subsequent phenotypic observations (such as EMT, migration, and invasion) stem from specific biological mechanisms rather than non-specific cytotoxic effects. Cells were exposed to the indicated concentrations of PAHs for 24 h. Vehicle controls containing medium with 0.8% DMSO (equivalent to the final solvent concentration in PAH-treated groups) were included in parallel.

### 2.2. Plasmid Construction and Cell Transfection

The wild-type LINC01376 sequence was amplified using primers listed in Table 1 and cloned into the AgeI and PstI restriction sites of the PGL-3 control vector (Invitrogen, Waltham, MA, USA). The recombinant plasmid was transformed into DH5α competent *E*. *coli* cells for amplification. Putative binding sites between LINC01376 and miR-15b-3p_R-1 were predicted using DIANA Tools (https://diana.e-ce.uth.gr/tools, accessed on 12 May 2023). A mutant version (LINC01376-MUT) lacking the predicted miR-15b-3p_R-1 binding site was generated by PCR-based site-directed mutagenesis. Wild-type LINC01376 (LINC01376-WT), mutant LINC01376 (LINC01376-MUT), and empty vector controls were transfected into NSCLC cells using Lipofectamine 2000 reagent (Mei5 Biotechnology, Beijing, China). Similarly, the 3′UTR of FGF2 containing either wild-type or mutated miR-15b-3p_R-1 binding site was amplified and cloned into the PGL-3 control vector (provided by Lanzhou University). Negative control siRNA, LINC01376-specific siRNA, FGF2-specific siRNA, miR-15b-3p_R-1 inhibitor, and miR-15b-3p_R-1 mimic (all from GenePharma, Shanghai, China) were separately transfected using Lipofectamine 2000.

### 2.3. Quantitative Real-Time PCR (qRT-PCR)

Total RNA was extracted from A549 and H1299 cells using TRIzol reagent (Thermo Fisher, Waltham, MA, USA), followed by chloroform phase separation, isopropanol precipitation, and washing with 75% ethanol. RNA pellets were dissolved in DEPC-treated water, and concentration and purity (A260/A280 ratio) were determined using a NanoDrop2000 spectrophotometer (Thermo Fisher, Waltham, MA, USA). Genomic DNA was removed, and cDNA was synthesized using the PrimeScript RT reagent Kit with gDNA Eraser (Takara, Otsu, Shiga, Japan) according to the manufacturer’s protocol: gDNA was eliminated at 42 °C for 2min, followed by reverse transcription at 37 °C for 15 min. cDNA was stored at 4 °C for short-term use or at −20 °C for long-term storage.

qRT-PCR was performed using TB Green Premix Ex Taq (Takara, Otsu, Shiga, Japan) on an iQ5 Optical Module PCR system (Bio-Rad, Hercules, CA, USA). The thermal cycling conditions consisted of an initial denaturation at 95 °C for 30 s, followed by 40–45 cycles of 95 °C for 5 s and 55–65 °C for 30 s, with a final extension at 95 °C for 15 s. Primer sequences are provided in Table 1. Glyceraldehyde-3-phosphate dehydrogenase (GAPDH) or U6 small nuclear RNA served as internal references. Melting-curve analysis confirmed the specificity of each PCR product. Relative RNA expression was calculated using the 2^−ΔΔCt^ method, with amplification efficiencies considered, and normalized to the respective reference gene.

### 2.4. Western Blot Assay

Proteins were extracted from treated or transfected A549 and H1299 cells using RIPA lysis buffer (Solarbio, Beijing, China). Equal amounts of protein were separated by 10% SDS-PAGE and transferred onto PVDF membranes (Millipore, Billerica, MA, USA). Membranes were blocked with 5% non-fat milk and incubated overnight at 4 °C with primary antibodies against E-cadherin, α-SMA, FGF2, GAPDH, and Vimentin (Cell Signaling Technology, Danvers, MA, USA). After washing, membranes were incubated with horseradish peroxidase-conjugated secondary antibodies for 1 h at room temperature. Protein bands were visualized using enhanced chemiluminescence (ECL) reagents (Bioscience, Shanghai, China) and quantified with Image-Lab (version 6.1) or Image-Pro Plus software (version 6.0).

### 2.5. Luciferase Reporter Assay

The dual-luciferase reporter system was used to validate targeting interactions between LINC01376 and miR-15b-3p_R-1 and between FGF2 and miR-15b-3p_R-1. Potential binding sites were predicted bioinformatically. Wild-type (LINC01376-WT and FGF2-3′UTR-WT) and mutant (LINC01376-MUT and FGF2-3′UTR-MUT) fragments were amplified by PCR using cDNA from A549 cells, digested with AgeI and PstI, and ligated into the PGL-3 control vector. Mutant constructs were generated using a site-directed mutagenesis kit. All plasmids were verified by restriction digestion and Sanger sequencing.

For reporter assays, 293T cells were seeded at 1 × 10^5^ cells/well in 12-well plates. After 12 h, cells were co-transfected using Lipofectamine 2000. For LINC01376 validation, cells were transfected with either miR-15b-3p_R-1 mimic or negative control (miR-NC), together with LINC01376-WT or LINC01376-MUT reporter plasmid and the pRL-TK Renilla luciferase control vector. An analogous setup was used for FGF2 validation with FGF2-3′UTR-WT or FGF2-3′UTR-MUT reporter plasmids. Forty-eight hours after transfection, cells were lysed with Passive Lysis Buffer (Promega, Madison, WI, USA). Luciferase activity was measured using the Dual-Luciferase Reporter Assay System (Promega, Madison, WI, USA) according to the manufacturer’s instructions. Firefly luciferase activity was normalized to Renilla luciferase activity for each sample.

### 2.6. Wound Healing Assay

Cell migration was assessed using a wound-healing assay. A549 and H1299 cells were seeded in 6-well plates at 3 × 10^5^ cells/cm^2^ and cultured for 12 h. After transfection and/or LMW-PAH treatment, a straight wound was created in each confluent monolayer using a sterile 200 μL pipette tip. Wells were gently washed with PBS to remove debris and then replenished with complete medium. Wound images were captured at 0 h and 24 h using an inverted microscope. Wound width was measured using ImageJ software (version win64.exe), and the relative wound closure rate was calculated as follows: (Wound area at 0 h − Wound area at 24 h)/Wound area at 0 h × 100%. Each experiment was performed in triplicate and repeated at least three times independently.

### 2.7. Transwell Invasion Assay

Cell invasion was evaluated using Transwell chambers (Corning, NY, USA) coated with Matrigel (abwbio, Shanghai, China). Briefly, 1 × 10^5^ cells in serum-free medium were added to the upper chamber. The lower chamber contained 600 μL of complete medium with 10% FBS as a chemoattractant. After 24 h incubation, cells remaining in the upper chamber were removed with a cotton swab. Invaded cells on the lower surface were fixed with methanol for 25 min, stained with 0.1% crystal violet for 25 min, washed, and air-dried. Cells in several fields were photographed and counted under a microscope.

### 2.8. Transcriptome Sequencing and ceRNA Network Construction

The transcriptomic data used for the discovery of LINC01376 in this study were generated and analyzed following a pipeline consistent with our previous report, as summarized below. Briefly, A549 cells were treated with 600 μM LMW-PAHs (Phe + Flu) for 24 h, after which total RNA was extracted. Strand-specific libraries depleted of ribosomal RNA and small RNA libraries were constructed and sequenced on the Illumina (Illumina, San Diego, CA, USA) NovaSeq 6000 (PE 150) and HiSeq 2000/2500 (SE 50) platforms, respectively. Raw sequencing data were subjected to quality control and aligned to the reference genome using HISAT2(version 2.1.0). Differential expression analysis was performed using edgeR, with thresholds set as |log2 FC| > 1 and *p* ≤ 0.05 for mRNAs and miRNAs, and log2 FC > 2 or <−1 with *p* < 0.05 for circRNAs. Target genes were predicted using TargetScan (v 5.0) and miRanda (v 3.3a), and ceRNA interaction networks were visualized with Cytoscape(version 3.7.1). Detailed experimental procedures and parameters have been described in our prior publication [34].

### 2.9. Statistical Analysis

The experimental results were analyzed using SPSS 26.0 software, with quantitative data presented as mean ± standard deviation. For normally distributed data, group comparisons were performed using one-way analysis of variance (ANOVA) if homogeneity of variance was satisfied, with post hoc pairwise comparisons conducted using the Least Significant Difference (LSD) method. In cases of unequal variances, Dunnett’s T3 method was applied for pairwise comparisons. For data that did not follow a normal distribution, the Kruskal–Wallis test was employed to assess differences among groups. A significance level of α = 0.05 was adopted, with *p* < 0.05 considered statistically significant. Graphs were generated using GraphPad Prism version 8.4.3.

## 3. Results

### 3.1. The Effects of LMW-PAHs on LINC01376 Expression

**Figure 1:**
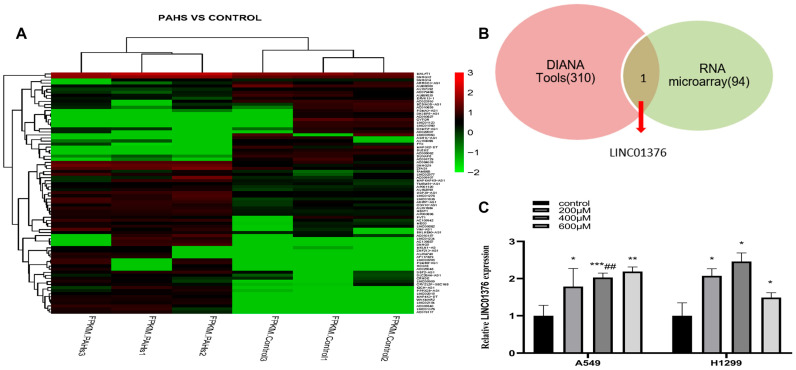
LncRNA profile based on microarray data and the expression of LINC01376 was upregulated in LMW-PAHs-induced A549 and H1299 cells: (**A**) Two-dimensional hierarchical clustering showed the differentially expressed lncRNAs profiles in 3 pairs of LMW-PAHs-treated A549 cells compared with the untreated A549 cells according to the microarray analysis. Red was higher expression levels and green was lower expression levels. Probes were in rows, and samples were in columns; (**B**) The Venn diagram displays the overlapping portion of lncRNAs between the sequencing data and the DIANA tool prediction outcomes, specifically LINC01376 indicated by the red arrow; (**C**) Expression of LINC01376 in A549 and H1299 cells, with different concentrations of LMW-PAHs (0, 200, 400, and 600 µM) detected by qRT-PCR. Significant differences are denoted as * *p* < 0.05, ** *p* < 0.01 and *** *p* < 0.001 compared with the control group (0 µg/mL LMW-PAHs treatment), and as ^##^ *p* < 0.01 compared with the 200 µM treatment group.

To further investigate the ceRNA mechanism underlying LMW-PAHs-induced EMT and based on previously established hsa_circ_0039929/hsa-miR-15b-3p_R-1/FGF2 regulatory axis, we first sought to explore whether other ncRNAs might also regulate FGF2 by sponging the same miR-15b-3p_R-1, thereby forming a more complex regulatory network. To this end, RNA sequencing was performed on three pairs of LMW-PAHs-treated and untreated A549 cells to identify differentially expressed lncRNAs. Compared with the control group, microarray analysis revealed 31 down-regulated and 64 up-regulated lncRNAs in LMW-PAHs-exposed A549 cells (Figure 1A). By integrating predictions from DIANA Tools with the sequencing data, we identified LINC01376 as a candidate lncRNA that was significantly upregulated (Figure 1B). Subsequent validation in both A549 and H1299 cells confirmed that LINC01376 expression was markedly increased upon LMW-PAHs exposure, with the most pronounced upregulation observed at 400 μM (*p* < 0.05; Figure 1C). Therefore, 400 μM was selected as the treatment concentration for subsequent tests.

### 3.2. The Effects of Knockdown of LINC01376 on EMT, Migration and Invasion

**Figure 2:**
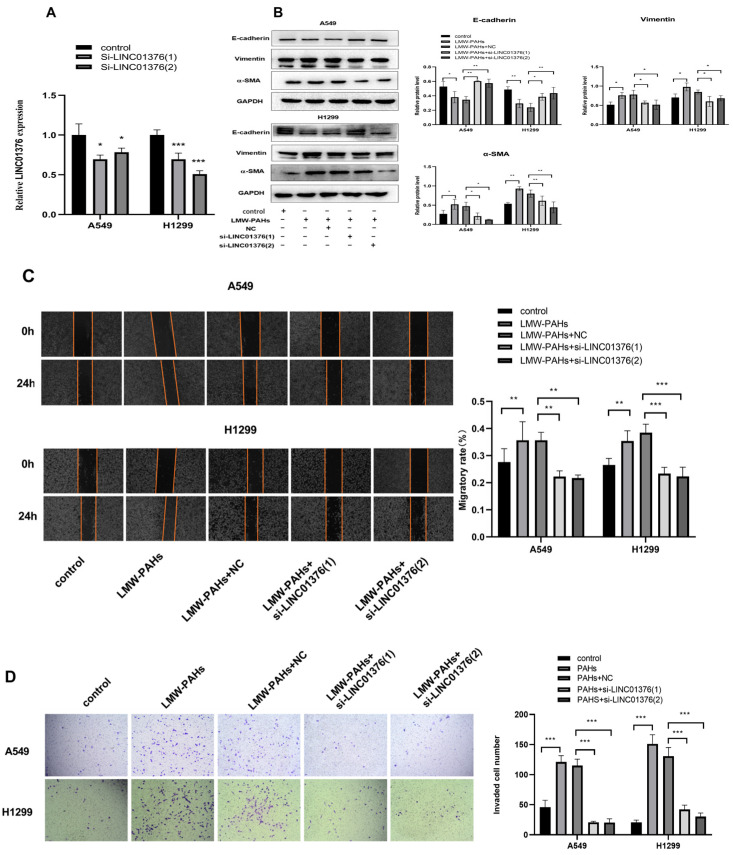
LINC01376 knockdown alleviated EMT, migration and invasion in LMW-PAHs-induced A549 and H1299 cells: (**A**) The efficiency of si-LINC01376 was analyzed via qRT-PCR; (**B**) The levels of EMT protein in LMW-PAHs-induced A549 and H1299 cells transfected with si-LINC01376 or NC were estimated by Western blot assay; (**C**) The migratory ability of LMW-PAHs-induced A549 and H1299 cells transfected with si-LINC01376 or NC by wound healing assay, the area between the two red line segments represents the wound area; (**D**) The cell invasion ability of LMW-PAHs-induced A549 and H1299 cells transfected with si-LINC01376 or NC by transwell invasion assay. * *p* < 0.05 and ** *p* < 0.01 and *** *p* < 0.001 compared to the corresponding negative control.

In this study, LINC01376 was silenced using LINC01376 siRNAs in LMW-PAHs-induced A549 and H1299 cells. First, qRT-PCR testing verified si-LINC01376′s knockdown effectiveness (Figure 2A). The Western blot results showed that LINC01376 promotes EMT in LMW-PAHs-induced A549 and H1299 cells because it resulted in a large increase in E-cadherin levels and a significant drop in Vimentin and α-SMA protein levels (*p* < 0.05) (Figure 2B). Additionally, this study investigated how LINC01376 affects invasion and migration. The knockdown of LINC01376 decreased the ability of cells to migrate (Figure 2C), and a transwell invasion experiment confirmed that the knockdown of LINC01376 impaired the ability of LMW-PAHs-induced A549 and H1299 cells to invade (Figure 2D). In light of the aforementioned findings, it may be concluded that LINC01376 enhanced EMT, migration, and invasion in LMW-PAHs-induced A549 and H1299 cells.

### 3.3. The Interaction Between LINC01376 and miR-15b-3p_R-1

**Figure 3:**
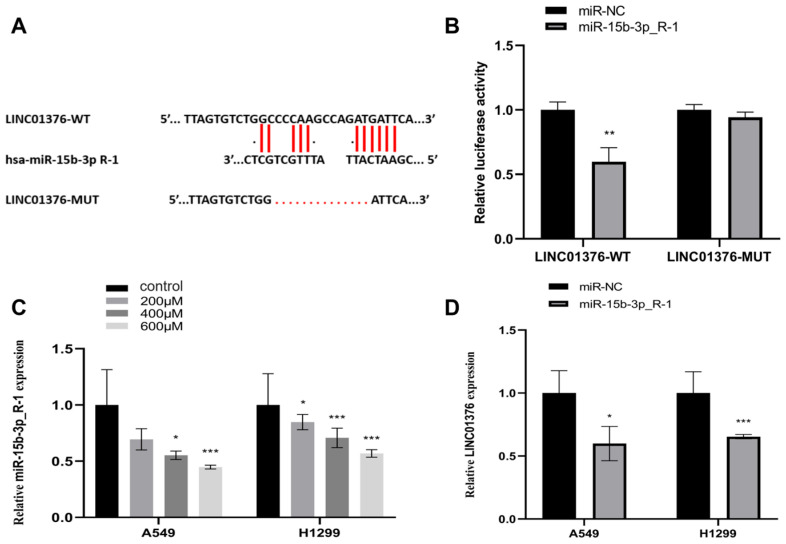
LINC01376 interacted with miR-15b-3p_R-1 in A549 and H1299 cells: (**A**) The binding sites between LINC01376 and miR-15b-3p_R-1 were predicted by DIANA Tools, the red line represents the binding sites and mutation sites between LINC01376 and miR-15b-3p_R-1; (**B**) The luciferase activity of LINC01376-WT or LINC01376-MUT in 293T cells co-transfected with miR-15b-3p_R-1 or miR-NC was examined by Dual-luciferase reporter assay. ** *p* < 0.01 compared with miR-NC group; (**C**) The mRNA level of LINC01376 in A549 and H1299 cells subjected to different concentrations of LMW-PAHs was determined by qRT-PCR. * *p* < 0.05 and *** *p* < 0.001 compared with the control group (0 µg/mL LMW-PAHs treatment); (**D**) The mRNA level of LINC01376 in A549 and H1299 cells transfected with miR-15b-3p_R-1 or negative control was tested by qRT-PCR. * *p* < 0.05 and *** *p* < 0.001 compared with miR-NC group.

The anticipated miR-15b-3p_R-1 binding location on LINC01376 was then used to generate a deletion mutation for a luciferase reporter assay (Figure 3A). It was demonstrated that miR 15b-3p interacted with LINC01376 at the anticipated binding location (Figure 3B) by reducing the luciferase activity of LINC01376-WT but not LINC01376-MUT when miR 15b-3p_R-1 was overexpressed. MiR-15b-3p_R-1 expression was downregulated in LMW-PAHs-induced A549 and H1299 cells, as shown in Figure 3C. Additionally, both cells overexpressing miR-15b-3p_R-1 showed a considerably lower level of LINC01376 than miR-NC (*p* < 0.05) (Figure 3D). The aforementioned findings confirmed LINC01376 and miR-15b-3p_R-1’s targeting affinity.

### 3.4. The Effects of miR-15b-3p_R-1 Overexpression on EMT, Migration, and Invasion

**Figure 4:**
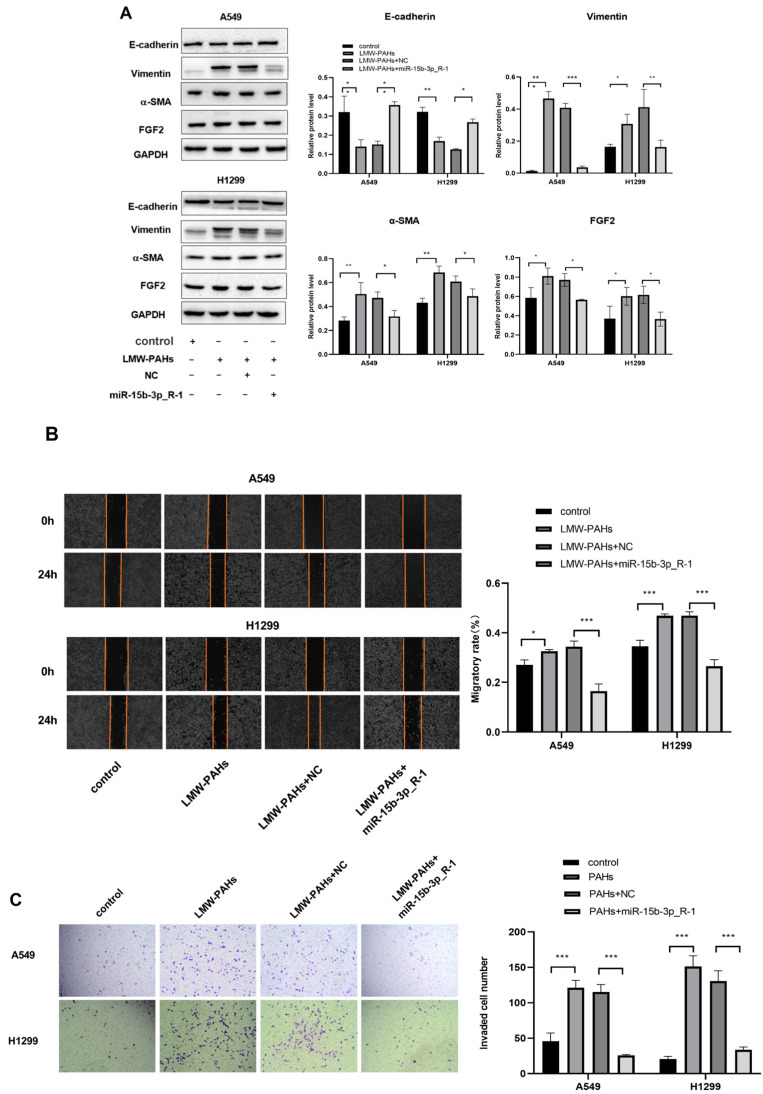
Effects of miR-15b-3p_R-1 overexpression on EMT, migration and invasion: (**A**) The levels of EMT protein and FGF2 in LMW-PAHs-induced A549 and H1299 cells transfected with miR-15b-3p_R-1 or NC were estimated by Western blot assay; (**B**) The migratory ability of LMW-PAHs-induced A549 and H1299 cells transfected with miR-15b-3p_R-1 or NC by wound healing assay, the area between the two red line segments represents the wound area; (**C**) The cell invasion ability of LMW-PAHs-induced A549 and H1299 cells transfected with miR-15b-3p_R-1 or NC by transwell invasion assay. * *p* < 0.05, ** *p* < 0.01 and *** *p* < 0.001 compared with the corresponding negative control.

Overexpression of miR-15b-3p R-1 in LMW-PAHs-treated A549 and H1299 cells increased E-cadherin levels and decreased Vimentin and α-SMA indicating suppression of EMT (Figure 4A). Overexpression of miR-15b-3p_R-1 EMT and reduced migrate and invade in LMW-PAHs-treated A549 and H1299 cells (Figure 4B,C). The results revealed overexpression of miR-15b-3p_R-1 decreased FGF2 protein levels and inhibited EMT, invasion, and migration in LMW-PAHs treated cells,. In addition, FGF2 protein levels were elevated by LMW-PAHs treatment while suppressed by miR-15b-3p_R-1 regulation of FGF2 (Figure 4A, *p* < 0.05).

### 3.5. The Interaction Between FGF2 and miR-15b-3p_R-1

**Figure 5:**
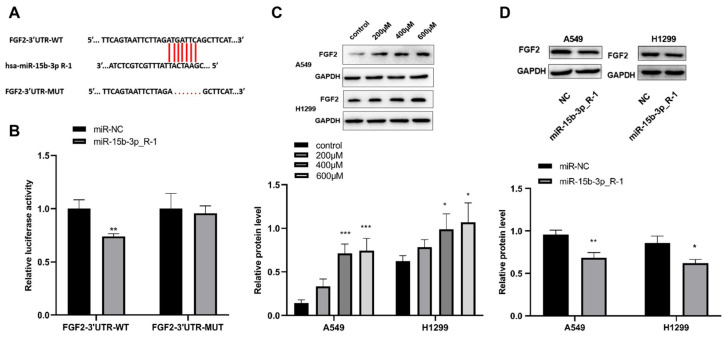
The direct interaction between FGF2 and miR-15b-3p_R-1: (**A**) The binding sites between miR-15b-3p_R-1 and FGF2 were predicted by the Targetscan database, the red line represents the binding sites and mutation sites between FGF2 and miR-15b-3p_R-1; (**B**) The luciferase activity of FGF2 3′UTR-WT or FGF2 3′UTR-MUT in 293T cells co-transfected with miR-15b-3p_R-1 or miR-NC was examined by Dual-luciferase reporter assay. ** *p* < 0.01 compared with miR-NC group; (**C**) The protein level of FGF2 in A549 and H1299 cells subjected to different concentrations of LMW-PAHs was determined by Western blot assay. * *p* < 0.05 and *** *p* < 0.001 compared with the control group (0 µg/mL LMW-PAHs treatment); (**D**) The protein level of FGF2 in A549 and H1299 cells transfected with miR-15b-3p_R-1 or miR-NC was tested by Western blot assay. * *p* < 0.05 and ** *p* < 0.01 compared with miR-NC group.

Figure 5A shows the miR-15b-3p_R-1 interaction sequences on FGF2 as well as the mutant sequences. After that, the mutant FGF2 3’UTR-MUT and the reporter plasmid FGF2 3′UTR-WT with the expected miR-15b-3p R-1 binding sites were decreased. As demonstrated in Figure 5B, the luciferase activity of FGF2 3′UTR-WT cotransfected with miR-15b-3p_R-1 was constrained in 293T cells, but that of FGF2 3′UTR-MUT cotransfected with miR-15b-3p_R-1 remained unaffected. In the meantime, Figure 5C shows that FGF2 is increased in LMW-PAHs-induced A549 and H1299 cells. Additionally, miR-15b-3p_R-1 overexpression reduced FGF2 protein levels in both A549 and H1299 cells (Figure 5D). The results from the aforementioned studies revealed that miR-15b-3p_R-1 might target FGF2 and suppress its production in A549 and H1299 cells exposed to LMW-PAHs.

### 3.6. The Regulation of LINC01376 on EMT, Migration and Invasion by miR-15b-3p_R-1/FGF2 Axis

**Figure 6:**
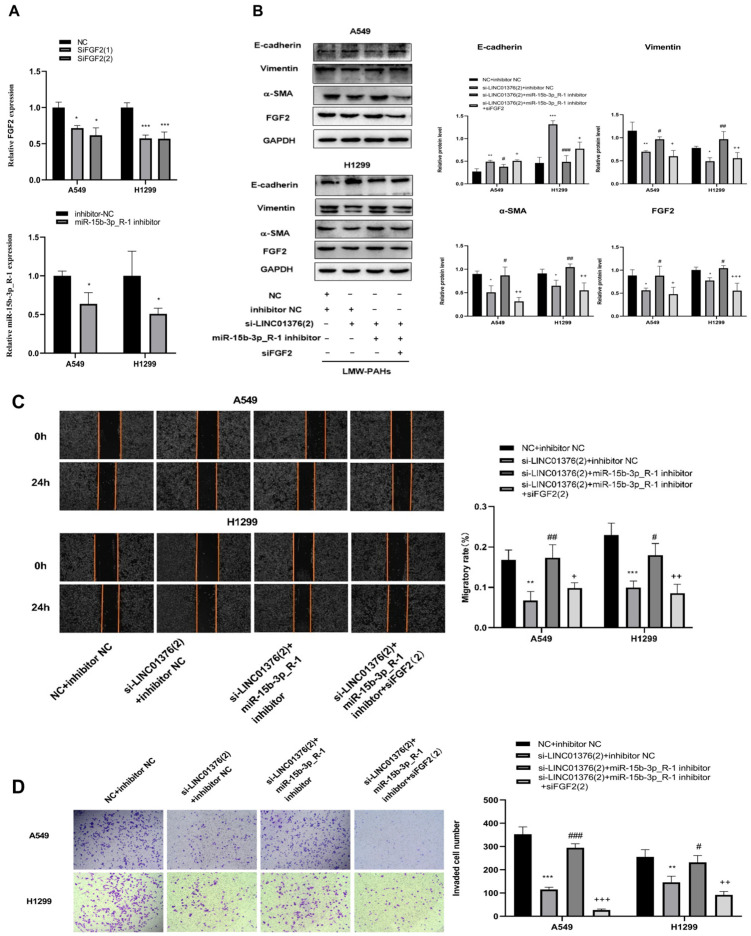
The regulation of LINC01376 on EMT, migration and invasion by miR-15b-3p_R-1/FGF2 axis: (**A**) The efficiency of FGF2 siRNA and miR-15b-3p_R-1 inhibitor was analyzed via qRT-PCR; (**B**) The levels of EMT protein and FGF2 were estimated by Western blot assay; (**C**) The migratory ability was assessed by wound healing assay; (**D**) The cell invasion ability was performed transwell invasion assay. * *p* < 0.05, ** *p* < 0.01, *** *p* < 0.001 compared with NC + inhibitor NC group; ^#^ *p* < 0.05, ^##^ *p* < 0.01, ^###^ *p* < 0.001 compared with si-LINC01376(2) + inhibitor NC group, and ^+^ *p* < 0.05, ^++^ *p* < 0.01, ^+++^ *p* < 0.001 compared with si-LINC01376 + miR-15b-3p_R-1 inhibitor + siFGF2(2) group.

Finally, whether LINC01376 controls EMT, migration, and invasion generated by LMW-PAHs via the miR-15b-3p_R-1/FGF2 pathway was established. To determine the effectiveness of the knockdown of FGF2 and miR-15b-3p_R-1 in A549 and H1299 cells, respectively, RT-qPCR was used in this work after the transfection of FGF2 siRNA and miR-15b-3p_R-1 inhibitor. According to Figure 6A’s RT-qPCR results, FGF2 and miR-15b-3p_R-1 was considerably downregulated when compared to NC (*p* < 0.05). Based on the outcomes of the earlier RT-qPCR tests, si-LINC01376(2) and siFGF2(2) were chosen for further testing. As predicted, si-LINC01376(2) significantly reduced the inhibitory effect of LMW-PAHs treatment on EMT, while miR-15b-3p_R-1 inhibitor significantly increased the expression of EMT-associated proteins (Vimentin and α-SMA levels) activated by LMW-PAHS and elevated E-cadherin protein levels (Figure 6B). FGF2 silencing further decreased Vimentin and α-SMA expression while increasing E-cadherin, and LINC01376 knockdown reduced LMW-PAHs-induced migration and invasion, similar to the effect of miR-15b-3p_R-1 overexpression (*p* < 0.05, Figure 6B). Furthermore, compared to the si-LINC01376 + miR-15b-3p_R-1 inhibitor group, siFGF2(2) further reduced the ability of LMW-PAHs-induced A549 and H1299 cells to migrate and invade (*p* < 0.05, Figure 6C,D). Therefore, it was discovered that LINC01376 sponges miR-15b-3p_R-1 to enhance FGF2 to promote EMT, migration, and invasion in LMW-PAHs-induced A549 and H1299 cells.

## 4. Discussion

Greenbury and Hay [38], who first described EMT, discovered that it is a process by which epithelial cells acquire mesenchymal properties. EMT is a complex molecular and cellular program. EMT in cells is characterized by the loss of cell contact and cell polarity breakdown, which reduces intercellular adhesion and increases cell motility, allowing them to move freely in the intercellular fluid [39]. EMT is connected to the progression of most cancers while causing the onset of cell migration and invasion, and EMT is the most significant pathway connected to malignant tumor invasion and migration [40]. This is because epithelial tumor cells can escape the primary tumor and invade new tissues and organs to form metastases. Studies conducted in vitro and in vivo have demonstrated that PM encourages the growth of EMT [41,42]. LMW-PAHs, a significant component of PM, have also been linked to EMT; however, research on the mechanisms by which LMW-PAHs induce EMT is currently lacking [43,44]. New research indicates that PAH-induced reactive oxygen species (ROS) can activate redox-sensitive pathways and modulate cellular metabolites, thereby fostering a microenvironment conducive to EMT and metastasis [12,45,46].

Growing evidence highlights the role of ncRNAs as miRNA sponges in post-transcriptional gene regulation [47]. Our prior work showed that hsa_circ_0039929 sponges hsa_miR-15b-3p_R-1 and attenuates EMT [34]. Building on this, we aimed to identify a lncRNA that could similarly sponge miR-15b-3p_R-1 during LMW-PAH-mediated EMT. The miR-15b-3p_R-1 expression was initially found to be suppressed, whereas LINC01376 was upregulated upon LMW-PAH exposure. Subsequent validation confirmed that LINC01376 functions as a ceRNA to sponge miR-15b-3p_R-1. Moreover, we demonstrated that FGF2 is a direct target of miR-15b-3p_R-1 and is transcriptionally activated during EMT. Collectively, the findings establish that in LMW-PAH-exposed A549 and H1299 cells, the LINC01376/miR-15b-3p_R-1/FGF2 axis accelerates EMT progression.

New research indicates that lncRNAs are involved in a variety of biological processes, including immune-related, cancerous, and EMT processes. To regulate gene expression, they are often utilized as ceRNA that sponge miRNAs [48,49,50]. However, only a few lncRNAs have been identified. As a result, our work initially predicted the identification of the lncRNA LINC01376 that binds to the miR-15b-3p_R-1 target. With supervised and unsupervised machine learning, gene coexpression networks, and enrichment analysis, prior research has shown that LINC01376 is a reliable biomarker for uterine corpus endometrial cancer (UCEC), demonstrating its elevated expression [51]. Furthermore, research has shown that small-cell lung cancer and obesity are both associated with an overexpression of LINC01376 [52]. However, it is still uncertain how LINC01376 contributes to the EMT brought on by LMW-PAHs. The results of the current investigation showed that in LMW-PAHs-induced A549 and H1299 cells, LINC01376 is upregulated and has a substantial affinity for miR-15b-3p_R-1. The reciprocal inhibition between LINC01376 and miR-15b-3p_R-1 was also experimentally validated. Functional tests verified that LINC01376’s knockdown EMT as well as cell migration and invasion in LMW-PAHs-induced A549 and H1299 cells, suggesting that LINC01376 may be a potential gene for lung EMT.

In many diseases, such as Alzheimer’s disease with rs363050 SNAP-25 GG homozygosity, microcystin-LR-induced hepatotoxicity, myocardial ischemia–reperfusion injury, and in 901/DDP cells and gastric adenomas, the expression of miR-15b-3p is downregulated [53]. MiR-15b-3p R-1 is downregulated in sequencing data and LMW-PAHs-treated A549 cells, suggesting that it may be implicated in the development of EMT produced by LMW-PAHs and play a partially disruptive function in this process, according to a prior study from our lab [36]. In addition, miR-15b-3p_R-1 overexpression inhibited the protein levels of FGF2, EMT, migration, and invasion, indicating that miR-15b-3p_R-1 may target FGF2 to regulate EMT, invasion, and migration in LMW-PAHs induced A549 and H1299 cells. This study confirmed that miR-15b-3p_R-1 expression was downregulated in LMW-PAHs-induced A549 and H1299 cells.

Furthermore, the study identified FGF2 as a target of miR-15b-3p_R-1. Basic fibroblast growth factor (bFGF), commonly known as FGF2, is a potent mitogenic factor that plays a role in cell proliferation, differentiation, anti-inflammation, angiogenesis, metabolism, and wound healing [54,55]. Upregulated FGF2 in pancreatic cancer may promote pancreatic cancer cell migration by increasing the expression of the downstream palladin protein through activation of the PI3K/AKT signaling pathway and EMT [56]. Additionally, FGF2 has been shown to activate the map kinase/mmp1 signaling pathway in malignant pleural mesothelioma cells, causing EMT [57]. In LMW-PAHs-induced A549 cells, we previously found that has-circ0039929 functions as a sponge for hsa-miR-15b-3p R-1 and reduces its effectiveness [32]. This upregulates the production of FGF2, activates the PI3K/AKT signaling pathway, and induces EMT. However, prior investigations did not directly demonstrate that miR-15b-3p_R-1 and FGF2 have a targeting connection. The current investigation thus confirmed this targeting link. Rescue studies revealed that the effects of LINC01376 + miR-15b-3p_R-1 suppression on LMW-PAHs-induced A549 and H1299 cells EMT, migration, and invasion were consistently reversed by FGF2 knockdown.

In summary, the study demonstrates that LINC01376 is upregulated in LMW-PAHs-induced A549 and H1299 cells, where it promotes EMT, migration, and invasion via the miR-15b-3p_R-1/FGF2 axis. The findings provide mechanistic insights into PAH-related toxicity, identify key regulators of EMT in lung cells, and suggest potential therapeutic targets for PAH-associated pathologies. Moreover, this work builds on our previous research and established a foundation for future in vivo and clinical investigations. Importantly, the systemic health impacts of environmental pollutants should be emphasized. Recent evidence indicates that exposures such as PM_2.5_ and heavy metals can significantly impair male reproductive health by altering sperm ncRNA profiles and nuclear basic proteins [58]. The biological effects of PAHs, including LMW-PAHs, may extend beyond their primary target organs are suggested. Future studies should investigate whether PAHs similarly affect germ cell function through analogous or distinct ncRNA regulatory networks, which will be critical for comprehensively assessing their health risks and informing protective strategies.

## 5. Limitations

While this study elucidates a novel ceRNA axis in LMW-PAH-induced EMT, several limitations should be acknowledged. First, the findings are based entirely on in vitro experiments using two NSCLC cell lines (A549 and H1299). The pathological relevance of the LINC01376/miR-15b-3p_R-1/FGF2 axis needs to be further validated in in vivo models and clinical samples from PAH-exposed populations. Second, the focused on a single regulatory axis; the potential crosstalk with other signaling pathways (e.g., TGF-β, Wnt) known to drive EMT warrants future investigation. Third, the LMW-PAHs exposure involved a defined mixture of phenanthrene and fluorene. The effects of other individual LMW-PAHs or more complex environmental mixtures containing both LMW- and HMW-PAHs remain to be determined. Addressing the limitations in future studies will enhanced the translational impact of our findings.

## 6. Conclusions

Based on the study, in A549 and H1299 cells, LMW-PAHs (Phe + Flu) promote EMT migration and invasion through the LINC01376 showing a significance upregulation at 400 μM exposure. Specifically, LMW-PAHs exposure upregulates LINC01376 expression and downregulates miR-15b-3p_R-1, leading to elevated expression of its target gene FGF2. Functional experiments demonstrated that knockdown of LINC01376 or overexpression of miR-15b-3p_R-1 significantly suppresses the aforementioned phenotypes. Mechanistically, LINC01376 acts as a ceRNA by directly binding to and “sponging” miR-15b-3p_R-1, thereby alleviating its inhibitory effect on FGF2 and establishing a complete ceRNA regulatory circuit. The findings not only elucidate a novel epigenetic regulatory mechanism by which LMW-PAHs drive EMT but also provide a theoretical basis for identifying potential biomarkers and therapeutic targets for PAH-associated pulmonary diseases. Future studies should validate the pathophysiological significance of this axis in vivo models and examine crosstalk with other key EMT signaling pathways, to support its translation into environmental health risk assessment and precision medicine.

## Figures and Tables

**Table 1:** Primers used in PCR and RT-qPCR assay.

Gene	Primer	Sequence
LINC01376	Forward	AAGTGGCTAACATCCGAGTTCATCATC
	Reverse	AGGCGTTGAATCATCTGGCTTGG
LINC01376-WT	Forward	ATAACCGGTGTATACATTCCTTCTCCCTTTG
	Reverse	GCACTGCAGGCATTCAATATTATTAGTGTTTGTAC
LINC01376-MUT	Forward	CTAAAGCACCCTCTAGTGTCTGGATTCAACGCCTGGAGAAGCTA
	Reverse	TAGCTTCTCCAGGCGTTGAATCCAGACACTAGAGGGTGCTTTAG
FGF2	Forward	CATCAAGCTACAACTTCAAGCA
	Reverse	CCGTAACACATTTAGAAGCCAG
FGF2 3′UTR-WT	Forward	GCGGAATTCGCTGCTTTATAGTTCTCTGGC
	Reverse	GCGCTGCAGAGAAATGAAAACTGACAGTAG
FGF2 3′UTR-MUT	Forward	AATAATTTCAGTAATTCTTAGAGCTTCATCATTAAGAATATC
	Reverse	GATATTCTTAATGATGAAGCTCTAAGAATTACTGAAATTATT
GAPDH	Forward	CAGGAGGCATTGCTGATGAT
	Reverse	GAAGGCTGGGGCTCATTT
hsa-miR-15b-3p_R-1_R-1	Forward	CGCGCGAATCATTATTTGC
	Reverse	AGTGCAGGGTCCGAGGTATT
	RT Primer	GTCGTATCCAGTGCAGGGTCCGAGGTATTCGCACTGGATACGACAGAGCA
U6	Forward	CTCGCTTCGGCAGCACA
	Reverse	AACGCTTCACGAATTTGCGT
	RT Primer	AACGCTTCACGAATTTGCGT

## Data Availability

The original contributions presented in this study are included in the article/Supplementary Materials. Further inquiries can be directed to the corresponding authors.

## Supplementary Materials

The following supporting information can be downloaded at: https://www.mdpi.com/article/10.3390/toxics14010054/s1.
