# Supplementary material for: The Role of the LINC01376/miR-15b-3p_R-1/FGF2 Axis in A549 and H1299 Cells EMT Induced by LMW-PAHs
Source: Toxics. 2026 Jan 6;14(1):54. doi: 10.3390/toxics14010054 (PMC12846051; doi:10.3390/toxics14010054)
Supplement: Supplementary file 1 [file toxics-14-00054-s001.zip › WB.pdf]

Electrophoretic gels and blots

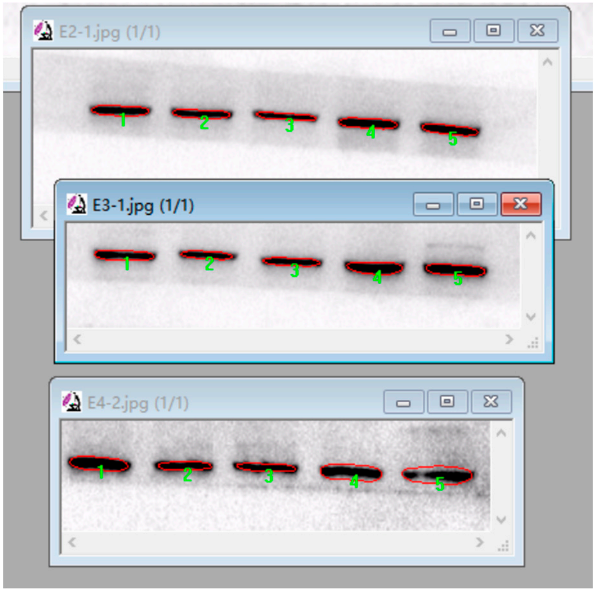

Figure 2. (B) The levels of E-cadherin protein in LMW-PAHs-induced A549 cells transfected with si-LINC01376 or NC were estimated byWestern blot assay.

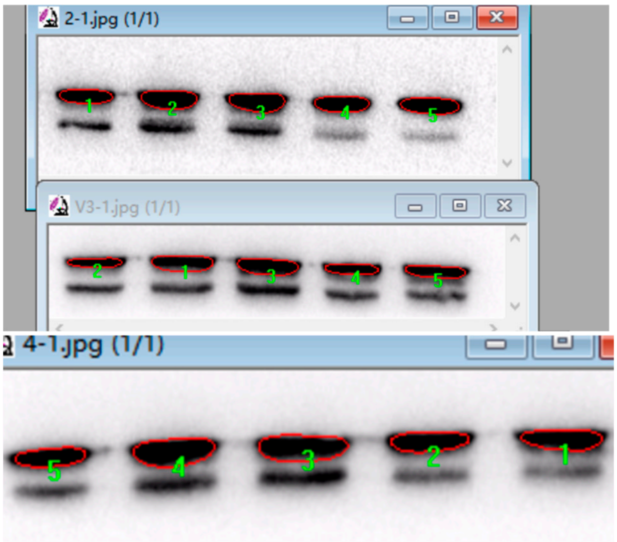

Figure 2. (B) The levels of Vimentin protein in LMW-PAHs-induced A549 cells transfected with si-LINC01376 or NC were estimated byWestern blot assay.

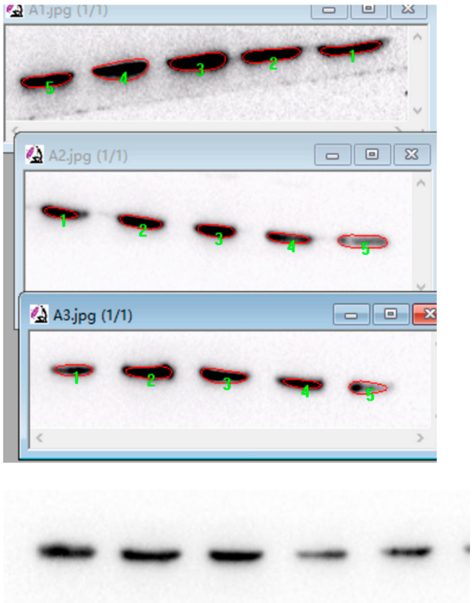

Figure 2. (B) The levels of α-SMA protein in LMW-PAHs-induced A549 cells transfected with si-LINC01376 or NC were estimated byWestern blot assay.

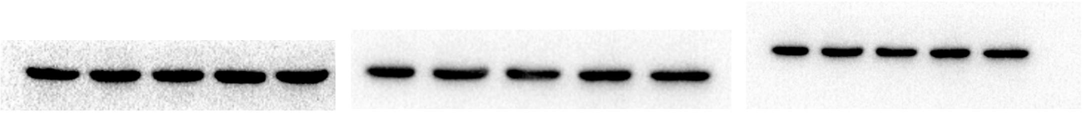

Figure 2. (B) The levels of GAPDH protein in LMW-PAHs-induced A549 cells transfected with si-LINC01376 or NC were estimated byWestern blot assay.

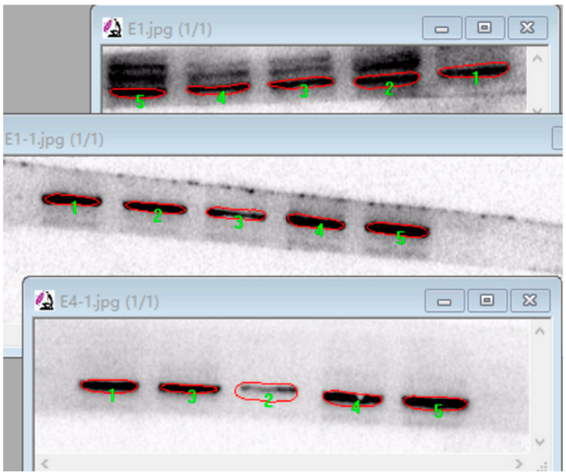

Figure 2. (B) The levels of E-cadherin protein in LMW-PAHs-induced H1299 cells transfected with si-LINC01376 or NC were estimated byWestern blot assay.

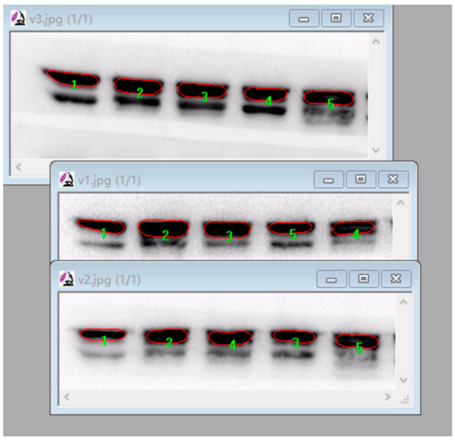

Figure 2. (B) The levels of Vimentin protein in LMW-PAHs-induced H1299 cells transfected with si-LINC01376 or NC were estimated byWestern blot assay.

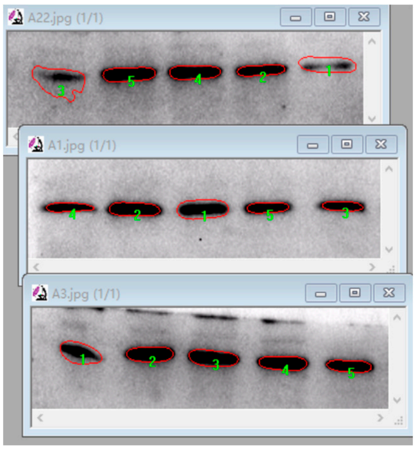

Figure 2. (B) The levels of α-SMA protein in LMW-PAHs-induced H1299 cells transfected with si-LINC01376 or NC were estimated byWestern blot assay.

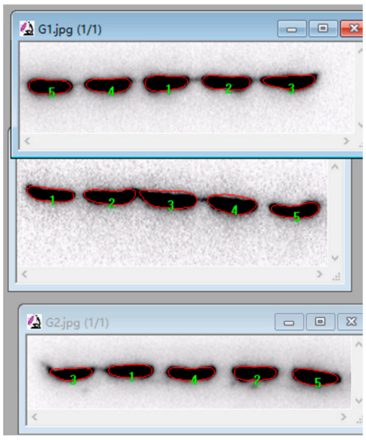

Figure 2. (B) The levels of GAPDH protein in LMW-PAHs-induced H1299 cells transfected with si-LINC01376 or NC were estimated byWestern blot assay.

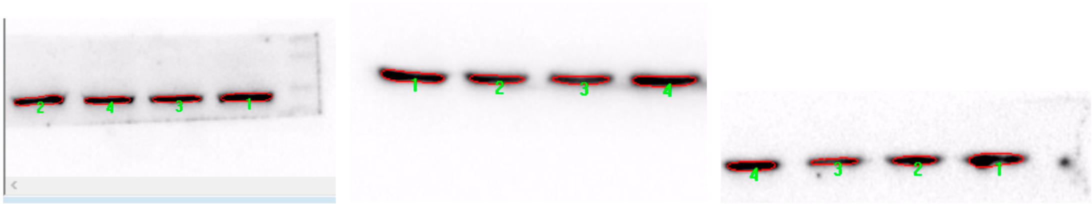

Figure 4. (A) The levels of E-cadherin protein in LMW-PAHs-induced A549 cells transfected with miR-15b-3p_R-1 or NC were estimated byWestern blot assay.

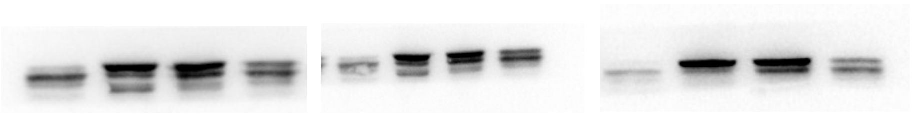

Figure 4. (A) The levels of Vimentin protein in LMW-PAHs-induced A549 cells transfected with miR-15b-3p_R-1 or NC were estimated byWestern blot assay.

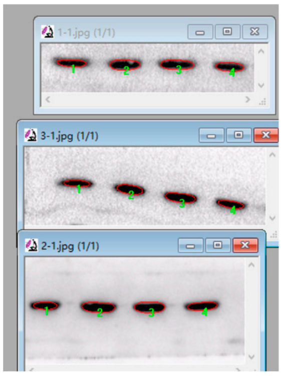

Figure 4. (A) The levels of α-SMA protein in LMW-PAHs-induced A549 cells transfected with miR-15b-3p_R-1 or NC were estimated byWestern blot assay.

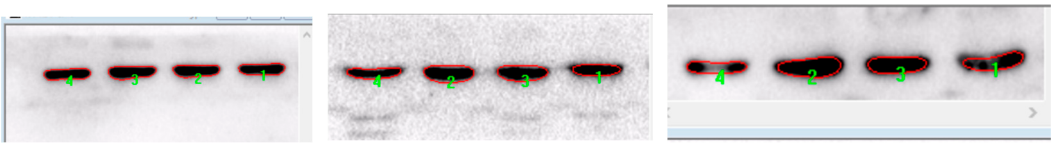

Figure 4. (A) The levels of FGF2 protein in LMW-PAHs-induced A549 cells transfected with miR-15b-3p_R-1 or NC were estimated byWestern blot assay.

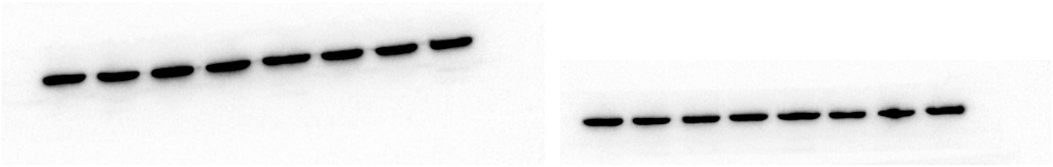

Figure 4. (A) The levels of GAPDH protein in LMW-PAHs-induced A549 cells transfected with miR-15b-3p_R-1 or NC were estimated byWestern blot assay.

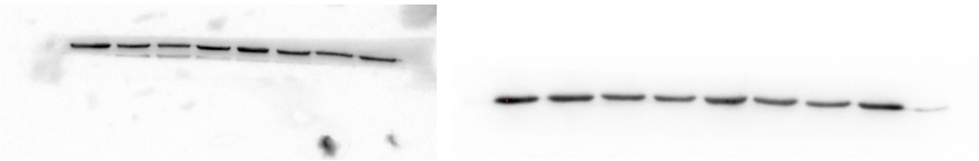

Figure 4. (A) The levels of E-cadherin protein in LMW-PAHs-induced H1299 cells transfected with miR-15b-3p_R-1 or NC were estimated byWestern blot assay.

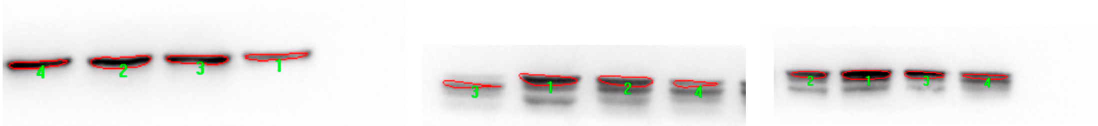

Figure 4. (A) The levels of Vimentin protein in LMW-PAHs-induced H1299 cells transfected with miR-15b-3p_R-1 or NC were estimated byWestern blot assay.

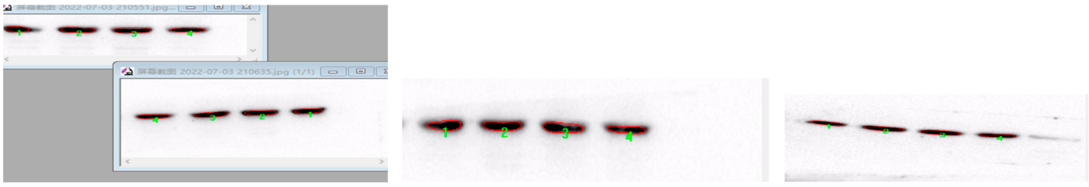

Figure 4. (A) The levels of α-SMA protein in LMW-PAHs-induced H1299 cells transfected with miR-15b-3p_R-1 or NC were estimated byWestern blot assay.

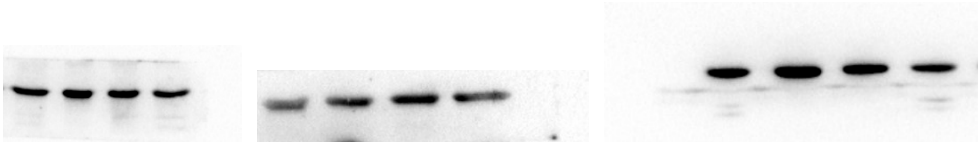

Figure 4. (A) The levels of FGF2 protein in LMW-PAHs-induced H1299 cells transfected with miR-15b-3p_R-1 or NC were estimated byWestern blot assay.

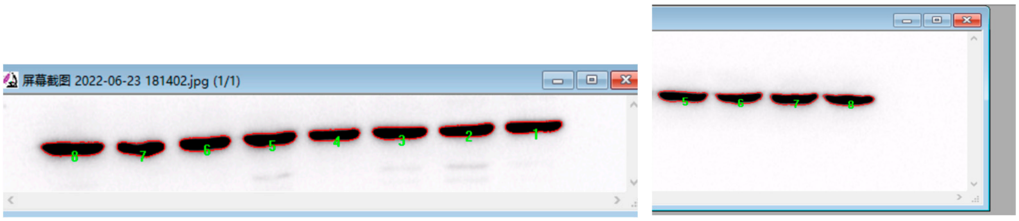

Figure 4. (A) The levels of GAPDH protein in LMW-PAHs-induced H1299 cells transfected with miR-15b-3p_R-1 or NC were estimated byWestern blot assay.

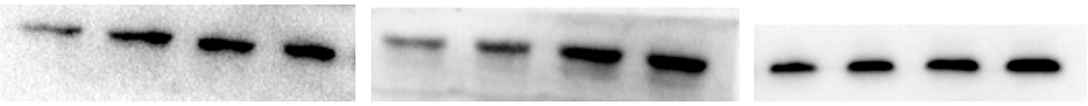

Figure 5. (C) The protein level of FGF2 in A549 cells subjected to different concentrations of LMW-PAHs was determined by Western blot assay.

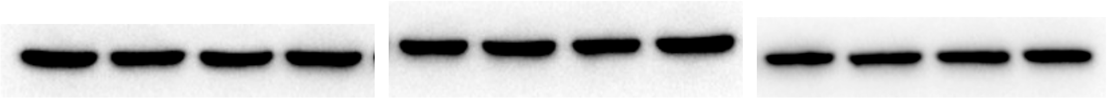

Figure 5. (C) The protein level of GAPDH in A549 cells subjected to different concentrations of LMW-PAHs was determined by Western blot assay.

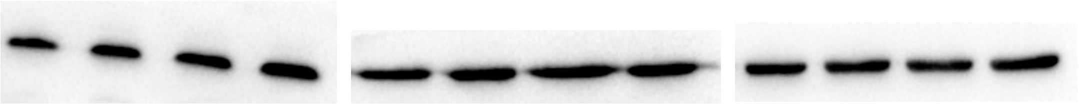

Figure 5. (C) The protein level of FGF2 in H1299 cells subjected to different concentrations of LMW-PAHs was determined by Western blot assay.

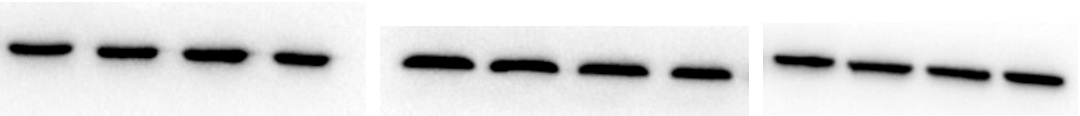

Figure 5. (C) The protein level of GAPDH in H1299 cells subjected to different concentrations of LMW-PAHs was determined by Western blot assay.

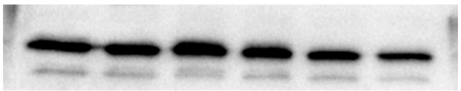

Figure 5. (D) The protein level of FGF2 in A549 cells transfected with miR-15b-3p_R-1 or miR-NC was tested by Western blot assay.

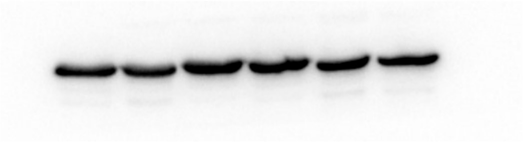

Figure 5. (D) The protein level of GAPDH in A549 cells transfected with miR-15b-3p_R-1 or miR-NC was tested by Western blot assay.

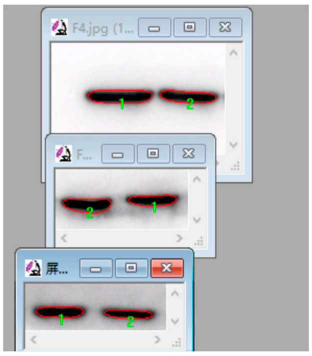

Figure 5. (D) The protein level of FGF2 in H1299 cells transfected with miR-15b-3p_R-1 or miR-NC was tested by Western blot assay.

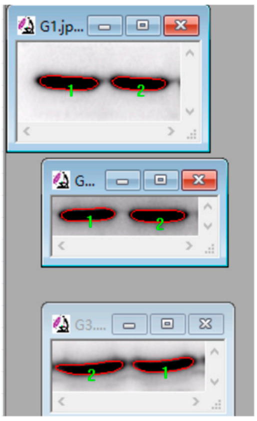

Figure 5. (D) The protein level of GAPDH in H1299cells transfected with miR-15b-3p_R-1 or miR-NC was tested by Western blot assay.

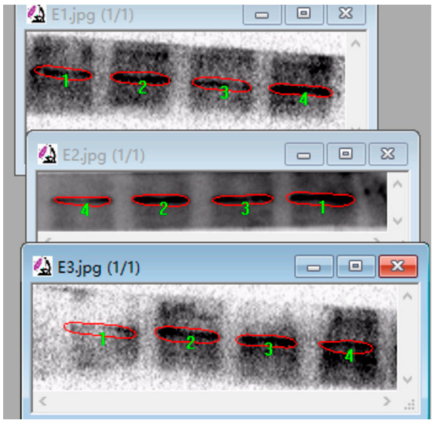

Figure 6. (B) The levels of E-cadherin protein in A549 cells were estimated by Western blot assay.

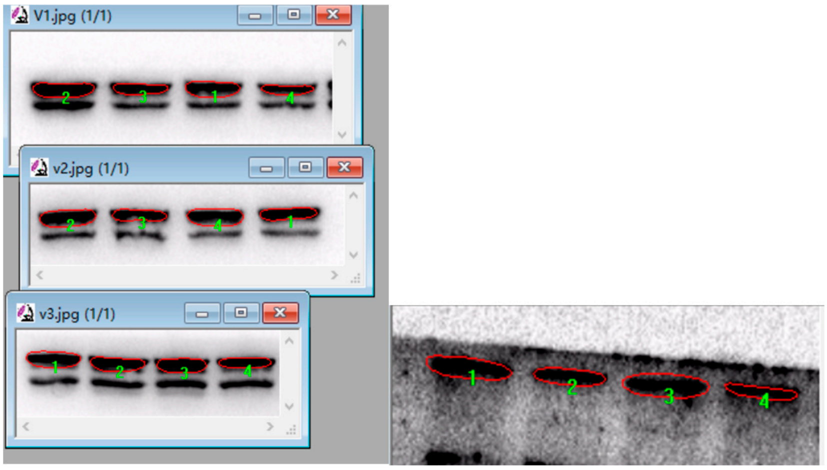

Figure 6. (B) The levels of Vimentin protein in A549 cells were estimated by Western blot assay.

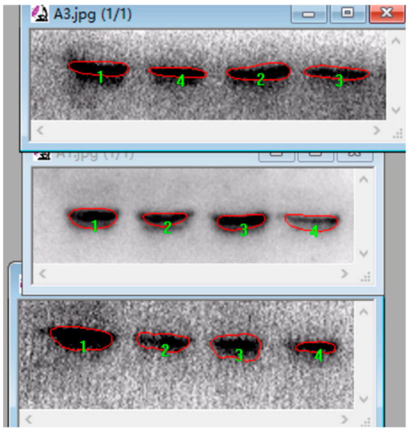

Figure 6. (B) The levels of α-SMA protein in A549 cells were estimated by Western blot assay.

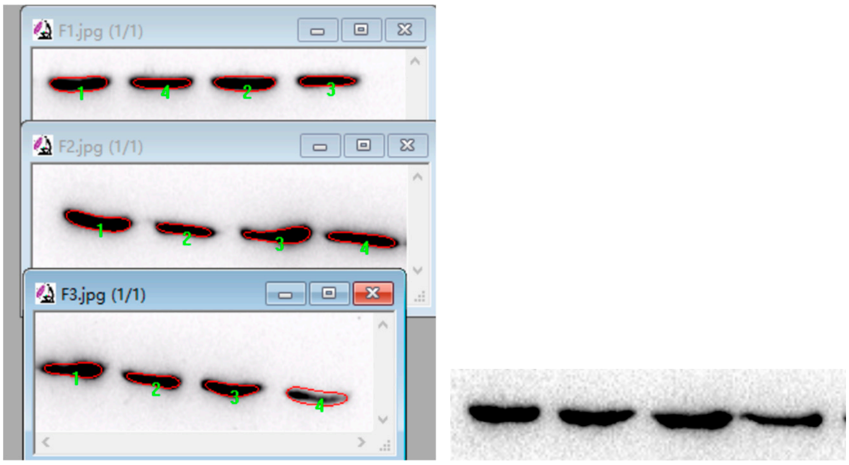

Figure 6. (B) The levels of FGF2 protein in A549 cells were estimated by Western blot assay.

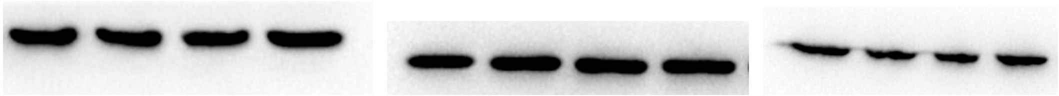

Figure 6. (B) The levels of GAPDH protein in A549 cells were estimated by Western blot assay.

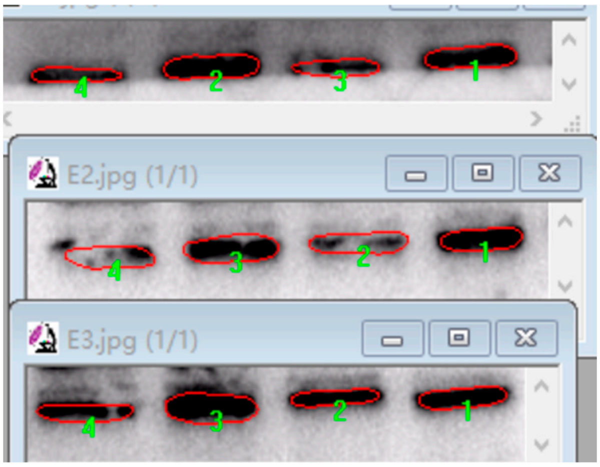

Figure 6. (B) The levels of E-cadherin protein in H1299 cells were estimated by Western blot

assay.

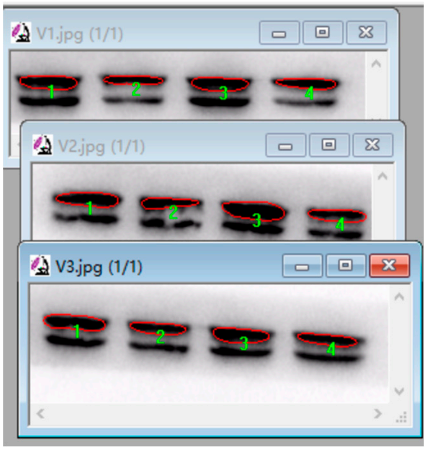

Figure 6. (B) The levels of Vimentin protein in H1299 cells were estimated by Western blot assay.

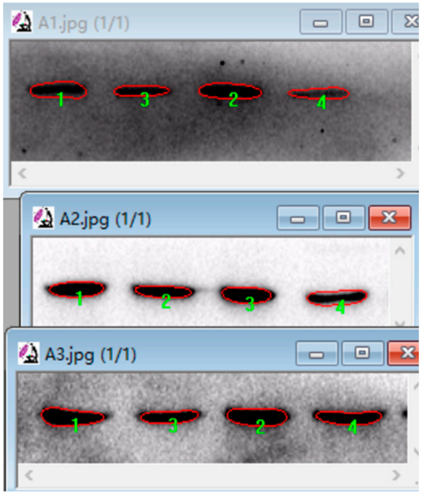

Figure 6. (B) The levels of α-SMA protein in H1299 cells were estimated by Western blot assay.

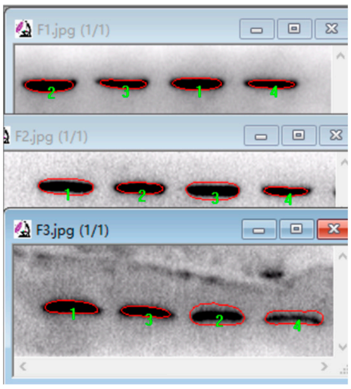

Figure 6. (B) The levels of FGF2 protein in H1299 cells were estimated by Western blot assay.

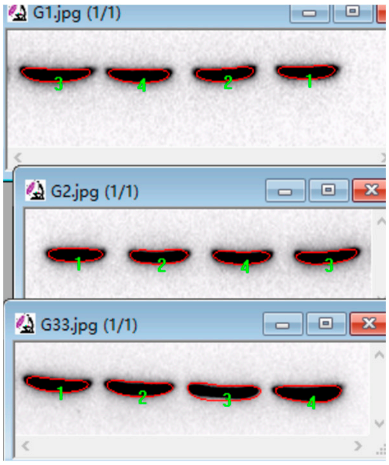

Figure 6. (B) The levels of GAPDH protein in H1299 cells were estimated by Western blot assay.
